# Genetics, Epigenetics, Cellular Immunology, and Gut Microbiota: Emerging Links With Graves’ Disease

**DOI:** 10.3389/fcell.2021.794912

**Published:** 2022-01-04

**Authors:** Fangyu Zhou, Xin Wang, Lingjun Wang, Xin Sun, Guiqin Tan, Wenwen Wei, Guangbing Zheng, Xiaomin Ma, Dan Tian, Hongsong Yu

**Affiliations:** ^1^ Department of Immunology, Special Key Laboratory of Ocular Diseases of Guizhou Province, Zunyi Medical University, Zunyi, China; ^2^ School of Basic Medical Sciences, Special Key Laboratory of Gene Detection and Therapy of Guizhou Province, Zunyi Medical University, Zunyi, China

**Keywords:** graves’ disease, pathogenesis, genetics, epigenetics, cellular immunology, gut microbiota

## Abstract

Graves’ disease (GD) is a well-known organ-specific autoimmune disease characterized by hyperthyroidism, goiter, and exophthalmos. The incidence of GD is approximately 2.0–3.0% in China and 0.5–2.0% in Western countries. Due to the complex pathogenesis and etiology of GD, current treatment methods have great side effects that seriously endanger human health. Therefore, it is particularly important to understand the pathogenesis of GD. Various studies have shown that genetics, epigenetics, cellular immunology, and gut microbiota are all involved in the development of GD. Genetically, *CD*25 gene and *VDR* gene polymorphisms are involved in the development of GD by increasing the ratio of Th17/Treg cells. Epigenetically, miR-23a-3p and lncRNA-MEG3 lead to Th17/Treg imbalance and participate in the progression of GD. Moreover, commensal microbe deletion can disrupt Th17/Treg balance and participate in the occurrence of GD. The imbalance of Th17/Treg cells induced by genetics, epigenetics, and gut microbiota plays a vital role in the pathogenesis of GD. Therefore, this article reviews the role of genetics, epigenetics, cellular immunology, and gut microbiota in the pathogenic mechanism of GD. This may lead to the development of novel therapeutic strategies and providing promising therapeutic targets.

## Introduction

Graves’ disease (GD), also known as toxic diffuse goiter, is one of the most common autoimmune thyroid diseases (AITDs). It is the main cause of hyperthyroidism, and hyperthyroidism syndrome, and the main clinical characteristics include varying degrees of goiter and exophthalmos (Wemeau et al., 2018). GD is believed to represent the autoimmune process of the thyroid, in which irritant autoantibodies combined with the thyroid-stimulating hormone receptor (TSHR) and activate thyroid function, leading to hyperthyroidism (Prabhakar et al., 2003). GD tends to occur in adult females aged 30–50, and the risk in women is six times higher than in men, with an annual morbidity of 20–50 cases per 100,000 people (Smith and Hegedus, 2016). The immunological characteristics of GD are the presence of thyroid-stimulating hormone receptor antibodies (TRAbs), thyroid peroxidase antibodies (TPOAbs), thyroglobulin antibodies (TgAbs), and other autoantibodies in the serum, leading to hyperthyroidism and diffuse thyroid enlargement (McLachlan and Rapoport, 2014).

GD is a T cell-mediated organ-specific autoimmune disorder. The infiltration of T lymphocytes in GD directly destroys the thyroid. Moreover, it can also stimulate B cells to differentiate into plasma cells that secrete antibodies (Li et al., 2019). Study has found that an increased number of CD4^+^ T cells and plasma cells were observed in GD patients (Ben-Skowronek et al., 2013). Naïve CD4^+^ T cells can differentiate into T helper 17 (Th17) and regulatory T cells (Tregs) when stimulated by specific antigens (Borst et al., 2018). A number of studies have shown that Th17/Tregs balance is vital to the pathogenesis of GD. Besides GD, Th17/Tregs balance may be also involved in the etiology of other autoimmune diseases, including rheumatoid arthritis (RA), psoriasis, multiple sclerosis, inflammatory bowel disease (Lee, 2018). Therefore, immune system alterations play an important role in the etiology of GD. In addition, genetics, epigenetics, and gut microbiota are also vital to the pathogenesis of GD.

Currently, three treatments are widely applied in clinical practice: radioiodine (RAI) therapy, antithyroid drugs (ATDs), and thyroidectomy. However, each method has side effects (Hu et al., 2020). Hence, the development of effective and specific treatments that can etiologically treat the disease is essential. Exploring the pathogenesis of GD may help to identify drug targets for novel therapies.

This article reviews the pathogenesis of GD from the aspects of genetics, epigenetics, cellular immunology, and gut microbiota. The aim is to provide a comprehensive overview of the basic principles to inform further development and clinical study of new drugs targeting the pathogenesis of GD.

## Cellular Immunological Mechanisms of GD

### Th1/Th2 Balance in GD

Stimulation by interleukin-12 (IL-12), interferon-γ (IFN-γ), and IL-2 and expression of the transcription factor T-bet induce naïve CD4^+^ T cells to differentiate into Th1 cells. This subset mainly synthesizes IL-1, IL-2, IFN-γ, and transforming growth factor beta (TGF-β), mainly through interactions with macrophages and other T lymphocytes. The presence of IL-4 suppresses the differentiation of naïve CD4^+^ T cells into Th1 cells and promotes the production of Th2 lymphocytes. GATA3 transcription factors are also implicated in Th2 development; this subset mainly synthesizes IL-4, IL-5, IL-6, IL-10, and IL-13. Th2 cells mainly interact with B and plasma cells, resulting in an increased production of antibodies and thereby mediating the humoral immune response (Eshaghkhani et al., 2016; Ramos-Levi and Marazuela, 2016).

Compared with controls, *T-bet* and *IFN-*γ mRNA levels in peripheral blood mononuclear cells (PBMCs) were prominently upregulated in GD patients, whereas *GATA*3 and *IL-4* mRNA expression levels were downregulated. In addition, a significant decrease in plasma IL-4 levels was observed in GD patients, while IFN-γ levels were higher in patients than in controls. These results suggest a Th1/Th2 imbalance in GD, which may be related to the pathogenesis of the disease (Eshaghkhani et al., 2016). Another study showed that the serum levels of IL-2 and IL-10 were elevated, while IFN-γ levels were lower in patients than in controls. IL-4 levels were not significantly different between patients and controls (Esfahanian et al., 2013). However, the levels of IFN-γ and IL-4 are discordant with the results of Eshaghkhani et al., and may be attributable to different sample types. In mouse models, IL-10 and IFN-γ expression levels were significantly increased (Ueki et al., 2011).

Taken together, the abnormal expression of Th1/Th2 cells and the abnormal secretion of related cytokines disrupts of Th1/Th2 balance. However, their expression in GD remains controversial, and additional studies on the role of Th1/Th2 balance in GD are warranted.

### Th17/Treg Balance in GD

Th17 cells are a subset of CD4^+^ T cells, primarily characterized by the generation of IL-17A, IL-17F, IL-21, and IL-22. The interaction of IL-1β, IL-6, and IL-23, the expression of the transcription factor retinoid-related orphan receptor gamma t (RORγt), and the activation of the STAT3 intracellular pathway play vital roles in the differentiation of Th17 cells (Tan et al., 2019). Treg lymphocytes are a heterogeneous population of lymphocytes characterized by their immunosuppressive function (Shao et al., 2018). Tregs act as negative regulatory cells through the synthesis of IL-10 and TGF-β (Tan et al., 2019). In recent years, the close relationship between Tregs and Th17 cells in GD has been explored in several studies (Li et al., 2016; Qin et al., 2017).

In Th17 cells and Tregs in PBMCs isolated from the peripheral blood of GD patients, the percentage of CD4^+^IL17^+^ T cells was significantly increased in GD patients compared with controls. In addition, the percentage of CD4^+^FoxP3^+^ Tregs was significantly reduced in GD patients in comparison to controls (Zhang et al., 2019). Analysis of Th17/Tregs ratio in the peripheral blood from GD patients indicated markedly lower ratios of CD4^+^IL17^+^/CD4^+^CD25^+^CD127^−^ and CD4^+^IL17^+^/CD4^+^CD25^+^CD127^−^FoxP3^+^ than in the controls. In untreated GD patients, a statistically significant positive correlation in CD4^+^IL17^+^/CD4^+^CD25^+^CD127^−^, CD4^+^IL17^+^/CD4^+^CD25^+^CD127^−^FoxP3^+^ T cells, and TRAb levels was observed. A positive association was also reported between the proportion of CD4^+^IL-17^+^ T cells and thyroid-stimulating antibody (TSAb) levels (Bossowski et al., 2016). In a mouse model of GD, the number of CD4^+^CD25^+^Foxp3^+^Treg cells decreased and the expression of IL-17 mRNA increased, but the CD4^+^IL-17^+^T cell subpopulation did not significantly differ from the controls (Yuan et al., 2017). Treg cells and the balance of Th17/Treg might be associated with the pathogenesis of GD, yet Th17 cells have not been shown to promote GD (Zhou et al., 2012). IL-17 and TGF-β were all upregulated in GD patients and mouse models (Yuan et al., 2017; Han et al., 2020).

In summary, inhibition of Treg cells disrupts the Th17/Treg balance, which ultimately leads to the occurrence and development of GD. In addition, Th17/Treg balance is vital to the pathogenesis of other autoimmune diseases (Noack and Miossec, 2014).

### Tfh Cells in GD

Follicular helper T (Tfh) cells have been identified as a new subgroup of effector helper T cells, which are vital to regulating the development of antigen-specific B cell immunity. Tfh cell differentiation is strictly regulated by the specific transcription factor Bcl-6, which is characteristically expressed in Tfh cells (Chen J et al., 2015).

An increasing body of evidence indicates that Tfh cells are significantly decreased in GD, which suggests that Tfh cells play an essential role in the pathogenesis of GD (Chen J et al., 2015; Wang et al., 2019). The proportions of effectors circulating Tfh (cTfh) and cTfh cell subsets (Tfh1, Tfh2, and Tfh17) in the peripheral blood of GD patients have been investigated. The proportion of effector cTfh cells and the Tfh2 subset were found to be increased in GD patients. Furthermore, a positive correlation between circulating Tfh2 (or PD-1^+^ Tfh) cells and serum TPOAb levels in GD patients has been reported (Liu et al., 2018). Tfhs and relevant factors in GD thyroid tissues were upregulated and protein expression levels of Tfh-related factors were also higher in GD thyroid tissues than in normal tissues (Zhang et al., 2015). These results imply that Tfh participates in the pathogenesis of GD.

These studies have revealed the abnormal expression of Tfh cells and related cytokines in GD patients. It shows that Tfh cells play a crucial role in the pathogenic mechanisms of GD and are thus potential therapeutic targets for GD. However, identification of the exact roles of Tfh cells in the pathogenesis of GD requires further investigation.

### Breg Cells in GD

Breg cells are immunosuppressive cells that support immune tolerance. Through the production of IL-10, IL-35, and TGF-β, Breg cells inhibit immunopathology and prohibit the expansion of pathogenic T cells and other pro-inflammatory lymphocytes (Rosser and Mauri, 2015).

The proportion of IL-10-producing B (B10) cells in the peripheral blood of GD patients was decreased and negatively correlated with TRAb levels. In addition, mouse disease models have generated similar results (Ji et al., 2020). These findings indicate that B10 cells suppress autoantibody production, and their abundance is decreased in GD patients. With the onset of GD, the proportion of CD19^+^CD1d^hi^CD5^+^ Breg cells in the PBMCs of Chinese patients is reduced (Qin et al., 2017). In addition, a conspicuous decrease in the number of CD19^+^CD24^hi^CD27^+^IL-10^+^ and CD19^+^IL-10^+^ B lymphocytes has been observed in untreated GD patients compared with controls (Rydzewska et al., 2018b).

In conclusion, Th1/Th2 balance, Th17/Treg balance, Tfh cells, and Breg cells contribute to the occurrence and development of GD. However, their precise roles in disease pathogenesis remain unclear. Additional studies on the cellular immunological mechanisms underlying GD to elucidate their interactions and provide new therapeutic strategies for GD are necessary.

## The Genetic Pathogenesis of GD

### The Association Between *HLA*-Related Gene Polymorphisms and GD Susceptibility

*HLA* is located on chromosome 6p21.3, has a total length about 4,000 kb, and contains a set of closely related genes, which are expressed on the cell surface as HLA-I and HLA-II molecules. These genes play important roles in antigen presentation to CD4^+^ T and CD8^+^ T cells (Xiaoheng et al., 2017). *HLA*-A, -B, -C, and -DRA are overexpressed in GD patients, with *HLA*-C showing the most significant upregulated expression (Yin et al., 2014).

*HLA-*I genes have been identified to be significantly associated with GD. In Iran, people with *HLA*-A*68 (case/control: 80/180, *p* = 0.004, OR = 4.23) and -B*08 (case/control: 80/180, *p* = 0.030, OR = 3.72) genes were susceptible to GD, while *HLA*-A*33 (case/control: 80/180, *p* = 0.011) appeared to play a protective role (Mehraji et al., 2017). A meta-analysis on the correlation between GD and *HLA*-B*46 in Asian populations revealed that *HLA*-B*46 was associated with an increased risk of GD (Li et al., 2013). In addition, the coexistence of *HLA*-B*46 (case/control: 73/159, *p* < 2.4 × 10^−8^, OR = 5.455) and -Cw*01 (case/control: 73/159, *p* < 0.00016, OR = 2.208) may be a genetic marker of early-onset AITD in Koreans. Moreover, the *HLA*-A*02 (case/control: 41/159, *p* < 0.014, OR = 1.905) was shown to confer susceptibility to GD, whereas -Cw*07 (case/control: 41/159, *p* < 0.001, OR = 0.144) played a protective role (Cho et al., 2011).

Among the *HLA*-II genes, the allelic frequency of *HLA*-DRB1*07 (case/control: 41/159, *p* < 0.015, OR = 0.128), -DQB1*0201 (case/control: 80/180, *p* = 0.040, OR = 0.50) and -DQA1*0201 (case/control: 80/180, *p* = 0.045, OR = 0.37) was found to be significantly lower among GD patients, whereas the frequency of *HLA*-DRB1*08 (case/control: 41/159, *p* < 5.6 × 10^−5^, OR = 3.436) was higher in GD patients than in controls (Cho et al., 2011; Mehraji et al., 2017). In a Romanian population, *HLA*-DRB1*03 (case/control: 77/445, *p* < 0.0001, OR = 3.29) and -DRB1*11 (case/control: 77/445, *p* = 0.045, OR = 1.70) were identified as the potential main susceptibility alleles of *HLA*-DRB1 related to GD. In addition, GD patients with *HLA*-DRB1*03/11 had a higher FT4/TT3 ratio and anti-TgAb levels, but *HLA*-DRB1*01 (case/control: 77/445, *p* = 0.007, OR = 0.20) and -DRB1*15 (case/control: 77/445, *p* = 0.038, OR = 0.42) were likely to have a protective function (Martin et al., 2014). Some *HLA* alleles were also associated with clinical features in GD patients, *HLA*-DRB1*1301 and -DQB1*0603 carriers more frequently develop larger goiters (Park et al., 2005).

In conclusion, *HLA* is the most diverse gene in the human genome, and its variation may be associated with the occurrence of GD or play a protective role. Hence, individuals carrying certain *HLA* alleles are susceptible to GD.

### The Association Between Non-*HLA* Gene Polymorphisms and GD Susceptibility

#### Association of T Cell-Related Gene Polymorphisms with GD Susceptibility

##### *CTLA*4 Gene Polymorphisms in GD

The *CTLA*4 gene is located on human chromosome 2q33 and has a total length of approximately 8.44 kb. It contains four exons and encodes 233 amino acids, generating two CTLA4 protein isoforms, namely, full-length CTLA4 (flCTLA4) and soluble CTLA4 (sCTLA4) (Fang et al., 2015). FlCTLA4 is expressed on the surface of the cellular membrane of activated T cells to play an immunosuppressive role. SCTLA4 may interfere with the coordinated stimulation signal and inhibit T cell proliferation (Patel et al., 2016).

The correlation between *CTLA*4 and the incidence of GD has been confirmed in multiple populations (Xiaoheng et al., 2017). In the Chinese Han population, the synergistic interactions of the *CTLA*4 SNPs, including rs231775 (case/control: 260/248, *p* = 0.002, OR = 1.521), rs231779 and rs3087243 (case/control: 260/248, *p* = 0.007, OR = 1.615), were associated with a significant increase in the risk of GD (Chen et al., 2018). Similarly, *CTLA*4/rs3087243 is also significantly associated with GD susceptibility in Kashmiri (case/control: 135/150, *p* < 0.001, OR = 2.21) as well as Brazilian (case/control: 282/308, *p* < 0.0001, OR = 2.593) populations (Shehjar et al., 2020; Bufalo et al., 2021). In addition, a significant association between *CTLA*4/rs231775 (case/control: 135/150, *p* < 0.001, OR = 1.85) and GD development has been reported in a Kashmiri population (Shehjar et al., 2020). Therefore, *CTLA4* polymorphisms are correlated to GD susceptibility. In patients with ophthalmopathy, the frequency of the rs231775 G allele was higher than in those without ophthalmopathy, suggesting that GD patients with *CTLA*4/rs231775 were more likely to develop ocular symptoms (Esteghamati et al., 2009).

##### *PTPN*22 Gene Polymorphisms in GD

*PTPN*22 is located on chromosome 1p13.3-13.1, consists of 16 exons, and encodes the 110-kDa lymphoid protein tyrosine phosphatase (LYP). LYP plays a negative regulatory role in T cell signaling (Xiaoheng et al., 2017).

With respect to the association between *PTPN*22 and GD susceptibility, the most studied SNP is rs2476601. The SNP rs2476601 is located in the N-terminal proline-rich motif and results in the substitution of arginine (Arg) with tryptophan (Trp) at codon 620 (Zhebrun et al., 2011). A significant association between *PTPN*22/rs2476601 (case/control: 171/200, *p* < 0.05, OR = 4.23) polymorphism and GD has been identified in a population from Northwest Russia and in Caucasians (Zhebrun et al., 2011; Luo et al., 2012). Moreover, female teenagers with GD in Poland have also exhibited a similar correlation, and allele A (case/control: 142/160, *p* = 0.009, OR = 2.13) was found to be an important risk factor (Rydzewska et al., 2018a). However, Bufalo et al. reported the opposite findings in Brazil (Bufalo et al., 2021). On the other hand, C allele of PTPN22/rs3789604 may be associated with an increased risk of liver damage in GD patients (Zhang et al., 2017).

##### *FoxP*3 Gene Polymorphisms in GD

The *FoxP*3 gene, which is located on the X chromosome, consists of 11 exons and encodes 431 amino acids. *FoxP*3 plays an essential role in the development and function of T cells, as well as differentiation into natural Tregs (Shehjar et al., 2018).

In a Caucasian population, the *FoxP*3 rs3761549 polymorphism was suggested to be involved in GD development in women (case/control: 109/75, *p* = 0.03, OR = 2.174) (Bossowski et al., 2014). In a Kashmiri population, the AC genotype (case/control: 135/150, *p* < 0.001, OR = 3.48) of rs3761548 and the TT genotype (case/control: 135/150, *p* < 0.001, OR = 5.62) of rs3761549 were considered risk factors of GD development (Shehjar et al., 2018). Similarly, in the Chinese Han population, allele A and genotype AA of rs3761548 were correlated with susceptibility to GD (Zheng et al., 2015).

##### *CD*25 Gene Polymorphisms in GD

*CD*25, also known as *IL-2RA*, is located on chromosome 10p15.1 and has a length of 60 kb. The expression of *CD*25 is a pivotal feature and phenotypic marker of Tregs (Brand et al., 2007).

It has been reported that *CD*25/rs2104286 (case/control: 650/1300; AA genotype: *p* = 8.772 × 10^−6^, OR = 1.636; A allele: *p* = 0.004, OR = 1.322) confers to GD susceptibility in the Chinese Han population (Du et al., 2021). The minor allele A (case/control: 1474/1609, *p* = 0.00017, OR = 1.43) of rs41295061 and the homozygous AA genotype (case/control: 1474/1609, *p* = 0.0053, OR = 1.54) of rs11594656 conferred susceptibility to GD in a Russian population (Chistiakov et al., 2011).

In summary, the expression in T cell-related genes may affect the differentiation and function of T cell subsets, and polymorphisms may lead to an imbalance of T cell subsets, thereby contributing to the occurrence of GD.

#### The Association Between B Cell Related Gene Polymorphisms and GD Susceptibility

##### *IKZF*3 Gene Polymorphisms in GD

The *IKZF*3 gene is found on chromosome 17q12-q21.1 in humans, contains nine exons, and is 104 kb in length. IKZF3 is a crucial transcription factor that inhibits the proliferation and differentiation of B cells (Li et al., 2018).

Li et al. were the first to report the relationship between *IKZF*3 polymorphisms and GD. They found that the association between the minor alleles of rs2941522 (case/control: 604/814, *p* = 0.02, OR = 1.21), rs907091 (case/control: 604/814, *p* = 0.006, OR = 1.25), rs1453559 (case/control: 604/814, *p* = 0.007, OR = 1.25), rs12150079 (case/control: 604/814, *p* = 0.006, OR = 1.29), and rs2872507 (case/control: 604/814, *p* = 0.004, OR = 1.27) and GD was significant (Li et al., 2018). More functional studies are needed to determine the contribution of *IKZF*3 polymorphisms to GD pathogenesis.

##### *BAFF* Gene Polymorphisms in GD

*BAFF* is located on chromosome 13q33, and plays an essential role in regulating the maturation, proliferation, differentiation and survival of B cells (Lin et al., 2016a).

Lin et al. found that serum BAFF levels in Chinese GD patients were higher than those of healthy controls. Further research showed that serum BAFF levels were significantly correlated with TSHRAb and anti-TPOAb levels only in women with active GD (Lin et al., 2016a). Lin et al. also studied SNPs in *BAFF* and showed that the G allele (case/control: 223/243, *p* = 0.009, OR = 0.70) of rs2893321 may decrease the risk of GD in women (Lin et al., 2016b). Lane et al. found that the frequency of another SNP (rs4000607) (case/control: 444/447, *p* = 0.019, OR = 1.80) was significantly different between GD patients and controls (Lane et al., 2019).

##### *CD*40 Gene Polymorphisms in GD

*CD*40 is located on chromosome 20q12-q13 and encodes a co-stimulatory receptor protein that plays an important role in B lymphocyte differentiation and antibody secretion. It is a member of the tumor necrosis factor receptor superfamily that has been associated with the pathogenesis of multiple autoimmune diseases (Wang D. et al., 2017).

With respect to the association between *CD*40 and GD susceptibility, rs1883832 is the most widely studied SNP. The CC genotype and the C allele with rs1883832 were associated with GD in the Japanese (case/control: 61/42; CC genotype: *p* = 0.041, OR = 2.438; C allele: *p* = 0.031, OR = 1.972) and Chinese populations (case/control: 196/122; CC genotype: *p* = 0.003, OR = 2.043; C allele: *p* = 0.008, OR = 1.57) (Inoue et al., 2012; Wang D. et al., 2017). Nevertheless, there was no significant association between *CD*40/rs1883832 and GD in the Brazilian and Pakistani populations (Mustafa et al., 2018; Bufalo et al., 2021). In addition, the G allele (case/control: 196/122, *p* = 0.001, OR = 5.472) of C64610G have been described as susceptibility factors for GD (Wang D. et al., 2017).

##### *BACH*2 Gene Polymorphisms in GD

*BACH*2 is located on chromosome 6q15, and encodes the BACH2 protein, which regulates the transformation of B cells into plasma cells. *BACH*2 is expressed in all PBMC isoforms, with the highest expression in B cells. A genome-wide association study (GWAS) via three stages verified that *BACH*2 is associated with GD in the Chinese Han population. *BACH*2/rs2474619 (case/control: 8882/9431, *p* = 3.28 × 10^−8^, OR = 1.13) is significantly associated with GD. However, there was no significant association between the rs2474619 genotypes and the level of *BACH2* gene expression (Liu et al., 2014).

##### *FAM*167*A-BLK* Gene Polymorphisms in GD

*FAM*167*A-BLK* is located on chromosome 8p23.1. The function of the FAM167A protein, also known as c8orf13, is unclear. BLK encodes a Src kinase that functions as a B cell signal transducer, is mainly expressed in B cells, and may influence the proliferation and differentiation of B cells. Song et al. found that the GG genotype of rs2618431 (case/control: 624/797, *p* = 0.04, OR = 1.246) may contribute to susceptibility to GD (Song et al., 2018).

In summary, B lymphocytes are the main effectors of antigen presentation and autoantibody production. B cell-related gene polymorphisms affect B cells, which are involved in the occurrence of GD.

#### Association Between Thyroid Hormone Related Gene Polymorphisms and GD Susceptibility

##### *TSHR* Gene Polymorphisms in GD

*TSHR* is located on chromosome 14q31 and encodes TSHR (Rydzewska et al., 2018a), a 764 amino acid polypeptide synthesized by a G protein-coupled receptor. It undergoes post-translational cleavage, producing TSHR A and B chains. The extracellular A subunit, which is shed, leads to the production of auto-antigens, facilitating the activation of non-self-tolerant CD4^+^ T cells. This ultimately results in the generation of stimulating antibodies (Hesarghatta Shyamasunder and Abraham, 2017).

The frequencies of the GG genotype (case/control: 180/111, *p* = 0.000105, OR = 2.602) and the G allele (case/control: 180/111, *p* = 0.0008, OR = 1.853) of rs4411444, the AA genotype (case/control: 180/111, *p* = 0.0228, OR = 1.740) of rs2300519, and the AA genotype (case/control: 180/111, *p* = 0.0052, OR = 1.984) and the A allele (case/control: 180/111, *p* = 0.0158, OR = 1.548) of rs179247 were higher in GD patients than in controls. The frequencies of these genotypes and alleles, as well as the rs2300519 A allele (case/control: 62/48, *p* = 0.0243, OR = 1.967) and the rs4903961 GG genotype (case/control: 62/48, *p* = 0.0147, OR = 2.588) and G allele (case/control: 62/48, *p* = 0.0166, OR = 2.061), were higher in patients with intractable GD than in controls and patients in GD remission (Fujii et al., 2017; Rydzewska et al., 2018a). In addition, rs179247 allele A was found significantly more frequently in GD patients without oculopathy than in patients with oculopathy, indicating that this allele is associated with a lower risk of GD in patients with oculopathy (Jurecka-Lubieniecka et al., 2014).

##### *TG* Gene Polymorphisms in GD

*TG*, which is located on chromosome 8q24, encodes a 660-kDa glycoprotein that supports the generation of thyroid hormones (Xuan et al., 2019). A significant relationship between rs2069550 (case/control: 436/316, *p* = 0.01, OR = 1.49) of the *TG* gene and GD has been reported (Gu et al., 2010).

In the Chinese Han population, rs2294025 (case/control: 9757/10626, *p* = 1.52 × 10^−9^, OR = 1.16) and rs7005834 (case/control: 9757/1062, *p* = 1.62 × 10^−7^, OR = 1.16) are two independent loci associated with susceptibility to GD (Xuan et al., 2019). Also, the frequency of the TT genotype (case/control: 131/89, *p* = 0.0283, OR = 0.484) of rs2703013 was significantly lower in GD patients than in controls. The distribution of the rs2958692 T allele (case/control: 50/40, *p* = 0.0055, OR = 2.382) was significantly different between patients with intractable GD and those with GD in remission (Mizuma et al., 2017).

In summary, thyroid hormone-related gene polymorphisms lead to the abnormal expression of related proteins. In addition, the production of autoantibodies leads to thyroid hormone secretion disorders, which are involved in the pathogenesis of GD.

#### The Association Between Apoptosis Related Gene Polymorphisms and GD Susceptibility

Apoptosis as a regulatory mechanism can remove excess unnecessary cells such as autoreactive lymphocytes. A lack of this ability causes the proliferation of cells that react with their own antigen, in turn leading to autoimmune diseases, including GD (Bossowski et al., 2008).

##### *VDR* Gene Polymorphisms in GD

*VDR* is located at chromosome 12q12-12q14 and consists of eight coding exons and 3 alternative 5′-noncoding exons spanning more than 75 kb of DNA (Meng et al., 2015). VDR is a nuclear receptor that controls the transcription of regulatory genes that are related to calcium metabolism and immune responses (Inoue et al., 2014).

Allele A (case/control: 417/301, *p* = 0.041, OR = 1.278) of rs7975232 has been associated with GD in the Chinese Han population (Meng et al., 2015). Similarly, the frequency of the C allele of *VDR*/rs7975232 was higher in patients with AITD (case/control: 255/76, *p* = 0.037, OR = 1.514), particularly GD patients (case/control: 139/76, *p* = 0.0349, OR = 1.594), than in controls (Inoue et al., 2014). Moreover, the A allele (case/control: 650/1209, *p* = 2.62 × 10^−2^, OR = 1.20) and AA genotype (case/control: 650/1209, *p* = 3.45 × 10^−4^, OR = 1.87) of rs7975232 were associated with a significant increase in susceptibility to GD. In GD patients with ophthalmopathy, the frequency of the rs7975232 AA genotype was higher than in those without ophthalmopathy, suggesting that GD patients with *VDR*/rs7975232 are more likely to develop ocular symptoms (Zhou et al., 2021). The frequency of the TT genotype of rs731236 was associated with the higher expression of VDR (Inoue et al., 2014). A meta-analysis found that the TT genotype (case/control: 2380/2235, *p* = 0.025, OR = 1.42) of rs731236 was associated with an increased risk of GD, but rs1544410 and rs7975232 of *VDR* were not different between GD patients and controls (Veneti et al., 2019).

##### *FAS* Gene Polymorphisms in GD

The *FAS* gene is located on chromosome 10q23. The receptor-ligand combination FAS-FAS ligand (FASL) is a chief mediator of apoptosis in many main physiologic processes. FAS and FASL both exist as membrane-bound and soluble types. The level of soluble FAS in subjects with the AA genotype of *FAS* -670 A/G was markedly higher than in those carrying the GG genotype (Mahfoudh et al., 2007).

There were no significant differences in rs2234767, rs1800682, and rs763110 between controls and AITD patients. However, serum soluble FASL levels in GD patients were significantly higher than in controls (Inoue et al., 2016).

##### *Bcl-*2 Gene Polymorphisms in GD

*Bcl-*2 is an anti-apoptosis factor, and the upregulated expression of Bcl-2 facilitates cell survival. In the *Bcl-*2/rs1800477 polymorphism, the frequency of the G allele (case/control: 264/79, *p* = 0.011, OR = 2.630), which is related to stronger anti-apoptotic function than the A allele, was markedly higher in AITD patients than in controls. No association between the polymorphism of the rs2279115 site and GD susceptibility has been observed (Inoue et al., 2016).

##### *TNFR*1 and *TNFR*2 Gene Polymorphisms in GD

The *TNFR*1 and *TNFR*2 genes, which encode TNF-RI and TNF-RII have been mapped to chromosomes 12p13 and 1p36, respectively (Glossop et al., 2005). TNF-RI possesses a death domain which can transduce the signal for cell death. While no differences in the genotype and allele frequency of *TNFR*1 rs2234649 have been observed, the G allele (case/control: 160/87, *p* = 0.038, OR = 1.827) of *TNFR*2 rs1061622 is associated with an increased risk of GD (Inoue et al., 2016).

##### *RNASET*2 Gene Polymorphisms in GD

*RNASET*2 is located on chromosome 6q27 and is the only RNase T2 family member in humans. *RNASET*2 plays a role in the initiation of human dendritic cells for the Th2 polarization of CD4^+^ T cells (Chen X J et al., 2015). There was a significant association between the risk allele G (case/control: 701/938, *p* = 0.005, OR = 1.225) of rs9355610 and a decrease in *RNASET*2 mRNA levels in a Chinese population (Chen et al., 2015). This association was also identified in the previous GWAS of GD (case/control: 5530/5026, *p* = 6.85 × 10^−10^, OR = 1.19) (Chu et al., 2011).

Genetic factors also play an important role in other autoimmune diseases. *HLA-DRB*1/rs13192471 confers a genetic predisposition to RA in a northeast Indian population (Das et al., 2018). *FoxP*3, *VDR*, *PTPN*22, *CTLA*4, and *CD*40 gene polymorphisms were associated with susceptibility to autoimmune diseases including RA, systemic lupus erythematosus, alopecia areata, and psoriasis (Wu et al., 2016; Bin Huraib et al., 2018; Hashemi et al., 2018; Moravvej et al., 2018; Fouad et al., 2019). With the extensive development of GWAS, many GD susceptibility genes have been identified that partly explain the clinical manifestations and pathogenesis of GD (Supplementary Table S1). However, due to differences in region, ethnicity, sample size, and genotyping methods, studies on the correlation between gene polymorphisms and GD susceptibility have generated inconsistent results. Therefore, it is necessary to increase sample sizes to elucidate the relationships between gene polymorphisms and GD susceptibility which can also provide reference for the identification of the susceptibility genes of other autoimmune diseases.

## Epigenetic Pathogenesis of GD

### The Role of DNA Methylation in GD

DNA methylation is a typical epigenetic modification (Wang B. et al., 2017). The methylation reaction is catalyzed by a cluster of enzymes, including DNA methyltransferases (DNMTs) (Picascia et al., 2015). The DNMT family includes DNMT1, DNMT2, DNMT3A, DNMT3B, and DNMT3L (Nawrocki et al., 2017). Among these, three enzymes are known to have DNMT activity: DNMT3a and DNMT3b are responsible for *de novo* methylation, while DNMT1 is required for maintaining DNA methylation (Jeltsch and Jurkowska., 2014). Compared to controls, the relative mRNA level of *DNMT*1 was significantly reduced in GD patients (Cai et al., 2015). Global hypomethylation and downregulation of *DNMT*1 mRNA expression in CD3^+^ T and CD19^+^ B lymphocytes in patients with newly diagnosed GD have also been observed, while there were no significant differences in DNMT3a and DNMT3b expression between GD patients and controls (Guo et al., 2018).

A genome-wide analysis of DNA methylation identified that 365 and 3322 CpG sites in CD4^+^ and CD8^+^ T cells are respectively differentially methylated in GD patients in comparison with controls. In addition, hypermethylation in the first intron of the *TSHR* gene has been associated with GD (Limbach et al., 2016). However, these findings are discordant with the results of Guo et al. on global hypomethylation patterns in CD3^+^ T and CD19^+^ B lymphocytes with GD patients, and may be attributable to different types of cells. Another study found a negative association between global methylation in B cells and serum TPOAb (Guo et al., 2018).

A statistically significant difference in methylation status intercellular adhesion molecule 1 (ICAM1) between GD patients and controls was observed, and there was a significant association between decreased *ICAM*1 methylation and exophthalmos in GD patients (Shalaby et al., 2019). Another study showed that DNA methylation decreased and DNA hydroxymethylation increased at the promoter of *ICAM*1, and this change was correlated with *ICAM*1 mRNA expression (Liu et al., 2017). Morita et al. assessed the methylation status of six CpG sites in the *TNFA* promoter region and found that the methylation level of the -72 CpG was notably higher in GD patients than in controls (Morita et al., 2018). Kyrgios et al. reported that *IL-2RA* gene promoter methylation status significantly differed among GD patients and controls, and the level of *IL-2RA* promoter DNA methylation was negatively correlated to anti-TPO and anti-TSI (Kyrgios et al., 2020).

Based on the above information, it can be hypothesized that abnormal DNA methylation is involved in the pathogenesis of GD. Nevertheless, it is still necessary to explore the abnormal DNA methylation of specific genes to provide new targets for the treatment of GD.

### The Role of Histone Modifications in GD

Chromatin is a dynamic structure that helps encapsulate the entire eukaryotic genome into the nucleus, as well as regulates DNA-related metabolic processes, including DNA transcription, recombination, repair, and replication. The basic unit of chromatin is composed of about 146 base pairs of DNA wrapped around a histone octamer (Li et al., 2007; Venkatesh and Workman, 2015; Martire and Banaszynski, 2020). Compared with GD patients carrying no chromosomal abnormalities, those with Turner- or Down-syndrome exhibited a prominent higher rate of transition from the previous Hashimoto’s hypothyroidism to Graves’ hyperthyroidism. This implies that chromosomal abnormalities and the coexistence of Hashimoto’s thyroiditis may increase the risk of developing GD. In addition, the dynamics of chromatin structure are strictly regulated by a variety of mechanisms including histone modification (Aversa et al., 2014). Histone modifications include acetylation, phosphorylation, methylation, ubiquitylation and SUMOylation (Bannister and Kouzarides, 2011). Mounting evidence has shown that abnormal histone modification profiles contribute to an imbalance in immune response, which in turn leads to the development of various autoimmune diseases (Araki and Mimura, 2017). In general, high levels of acetylation and trimethylation of the Lys-4 residue of histone 3 (H3K4me3) were detected in the promoter regions of transcriptionally active genes in individuals with GD (Stefan et al., 2011).

Genome-wide analysis of DNA methylation in CD4^+^ and CD8^+^ T cells revealed that hypermethylation was related to reduced levels of H3K4me3 and H3K27ac marks in several T cell signaling genes. However, these results were discordant to the findings of Guo et al. on global hypomethylation in CD3^+^ T and CD19^+^ B lymphocytes of GD patients, which may be attributed to the use of different types of cells. Regions with lower H3K4me3 or H3K27ac signals were related to hypermethylated CpGs and areas with strong H3K4me3 or H3K27ac signals overlapping with hypomethylated CpGs. In addition, gene ontology and pathway analysis have suggested reduced H3K4me3 and H3K27ac signals in genes involved in T cell activation (Limbach et al., 2016). In PBMCs, global histone H3K9 methylation was significantly downregulated in GD patients compared with controls. Global H3K4 methylation was decreased in GD patients, but this difference was not statistically significant (Yan et al., 2019).

Yan et al. reported that the mRNA expression of histone deacetylases HDAC1 and HDAC2 in the PBMCs of GD patients was significantly higher than that of controls. This indicates that histone acetylation modifications are abnormal in PBMCs of GD patients (Yan et al., 2015).

In summary, histone expression is associated with methylation status and histone modifications of the genes that may regulate its expression. It is obvious that histone modifications influence the occurrence of GD. However, studies on the role of histone modification in GD are limited, and more investigations are needed to elucidate its role in GD.

### The Role of miRNAs in GD

MicroRNAs are a class of small non-coding RNAs that negatively control gene expression by directly combining with the 3′ untranslated region of their mRNA targets (Mehta and Baltimore, 2016). MicroRNAs play a crucial regulatory role in Toll-like receptor signaling, resulting in the activation of the NF-κB, IRF, and AP-1 transcription factors, which regulate the expression of pro-inflammatory cytokines (Wu et al., 2015).

The effects of miRNAs in GD are described below. MiR-181d was found to be upregulated in GD patients and miR-346 was significantly downregulated. Furthermore, miR-346 regulates CD4^+^CXCR5^+^ T cells by acting on Bcl-6, a positive regulator of Tfh cells (Chen J et al., 2015). The expression level of miR-146a in plasma was significantly reduced in AITD patients in comparison with controls. However, the expression level of miR-146a in PBMCs was increased. The reason of discordant expression levels in miR-146a in plasma and PBMCs might be that miR-146a inhibits the release of miR-146a from PBMC into plasma, and thus inducing the excessive inflammation in the thyroid of AITD patients. Thus miR-146a may be related to the occurrence of AITD (Otsu et al., 2017). MiR-155 enhances Treg and Th17 cells differentiation by targeting SOCS1 (Yao et al., 2012). However, another study showed that overexpression of miR-155 results in increased Th1 differentiation. This result is complementary to the observed bias toward Th2 differentiation in CD4^+^ T cells lacking miR-155, thereby contributing to the regulation of Th1/Th2 balance (Banerjee et al., 2010). The expression of miR-154, miR-376b, and miR-431 was suppressed in the PBMCs of initial GD patients (Liu et al., 2012). In comparison to GD patients in remission, circulating miR-23b-5p and miR-92a-39 levels were reduced in intractable GD patients, whereas let-7g-3p and miR-339-5p were increased (Hiratsuka et al., 2016). The serum levels of miR-16, miR-22, miR-375, and miR-451 were increased in GD patients compared with controls (Yamada et al., 2014). MiR-23a-3p levels were significantly downregulated in GD patients. Animal experiments have demonstrated that miR-23a-3p overexpression strengthens Tregs function *in vivo* (Zhang et al., 2019). Yao et al. discovered five miRNAs that were differentially expressed between GD patients and controls. There were four upregulated miRNAs (has-miR-122-5p, 16-1-3p, 221-3p, and 762) and one downregulated miRNA (has-miR-144-3p) (Yao et al., 2019). Qin et al. reported that miR-22 and miR-183 were overexpressed in GD patients, but miR-101, miR-197, and miR-660 levels were downregulated in the thyroid tissue of GD patients (Qin et al., 2015).

Martínez-Hernández et al. indicated that miRNAs are associated with autoimmune antibody levels in GD. They found that miR-let7d-5p was negatively correlated to TPOAb levels, while miR-21-5p and miR-96-5p were positively correlated with TPOAb, TgAb, and TRAb levels. MiR-142-3p and miR-301a-3p were only positively associated with TRAb levels, and miR-6503-3p was correlated with TPOAb levels (Martinez-Hernandez et al., 2018).

The above studies indicated that miRNAs participate in the regulation of gene and autoimmune antibody expression, ultimately contributing to the occurrence and development of GD and may hence serve as biomarkers for the diagnosis, treatment, and prognosis of GD.

### The Role of lncRNAs in GD

Long non-coding RNAs (LncRNAs) are non-coding RNAs with lengths of >200 nt that are transcribed by RNA polymerase II. Most lncRNAs are transcribed from the 5′ end to 3′ end of genes, but lncRNAs do not encode proteins (Ponting et al., 2009).

LncRNAs have a wide range of effects and are involved in transcriptional regulation, post-transcriptional regulation, and participate in chromatin remodeling and protein transport (Quinn and Chang, 2016). LncRNAs can be used as miRNA sponges that control the expression of target genes in various physiopathological processes (Zhang et al., 2018). Qiu et al. found that lncRNA-MEG3 may serve as a competing endogenous RNA that regulates RORγt expression via miR-17, thus affecting Th17/Treg balance. This indicates that the lncRNA/miRNA axis plays an important role in the etiology of GD (Qiu et al., 2019). The level of the RNA transcript Heg in monocytes is negatively correlated to TRAb levels in untreated GD patients (Christensen et al., 2008). However, Christensen et al. found that a decrease in TRAb levels in treated GD patients was not correlated to changes in Heg RNA levels (Christensen et al., 2011). These results suggest that Heg is related to the pathogenesis of GD.

Studies on the role of epigenetic factors in the development of GD have improved our understanding of the pathogenic mechanisms of GD (Supplementary Table S2). In parallel, epigenetics also play a pivotal role in other autoimmune diseases (Long et al., 2016; Karagianni and Tzioufas, 2019). Understanding the role of epigenetics in GD allows the generation of new ideas on the pathogenesis of other autoimmune diseases. Therefore, further studies are needed to: 1) elucidate the relationship between epigenetic factors and GD, 2) explore the causes and mechanisms of epigenetic changes, and 3) identify effective molecular targets for the diagnosis, treatment and prognosis of GD.

## The Gut Microbiota and GD

The human microbiome is an essential but largely ignored part of the human genome (Grice and Segre, 2012). The collection of bacteria, archaea, and eukarya colonizing the human gastrointestinal (GI) tract is called the “gut microbiota” which has coevolved with the host for thousands of years to form a complex and mutually beneficial relationship. The microbiota brings many benefits to the host, through a series of physiological functions. Microbial composition disruption is known as dysbiosis. With the development of increasingly sophisticated methods for analyzing and characterizing complex ecosystems, the role of microbiota in a variety of intestinal and extraintestinal diseases has become increasingly clear (Thursby and Juge, 2017).

An analysis of the relative richness and diversity has indicated that the diversity of intestinal bacteria in GD patients was lower than in controls. The microbiota in GD patients mainly included four phyla (Firmicutes, Bacteroidetes, Proteobacteria, and Actinobacteria). One study showed that in GD patients, the abundance of Bacteroidetes and Proteobacteria was higher and the level of Firmicutes was lower than in controls (Ishaq et al., 2018). Moreover, another study revealed that the relative abundance of Bacteroidetes and Actinobacteria increased and Firmicutes decreased in GD patients compared with healthy controls (Chang et al., 2021). In the Han population of Southwest China, GD patients showed a significantly higher abundance of Firmicutes, Proteobacteria, and Actinobacillus. Additionally, the proportion of Firmicutes was significantly higher in GD patients than in controls, while the proportion of Bacteroidetes was significantly lower in GD patients than in controls (Yang et al., 2019). However, another study on the Han population of Shanghai China found that the proportion of Firmicutes in GD patients was significantly reduced, while the proportion of Bacteroidetes was significantly increased (Jiang et al., 2021). The reason for these opposite results may be that two studies collected samples from different regions and had small sample sizes (case/control: the former 15/15; the latter 46/59).

In the genus level, the abundance of *Lactobacillus* is higher in GD in several studies, which suggests *Lactobacillus* may be important to the pathogenesis of GD. The abundance of the genera *Oribacterium*, *Lactobacillus*, *Aggregatibacter*, and *Mogibacterium* was significantly higher in GD patients than in controls (case/control: 15/15, *p* < 0.05) (Yang et al., 2019). Furthermore, GD patients have higher counts of *Bacteroides* and *Lactobacillus* (case/control: 45/59, *p* < 0.05) (Jiang et al., 2021). Chen et al. found that the relative abundance of *Lactobacillus*, *Veillonella* and *Streptococcus* were significantly higher in GD (case/control: 15/14, *p* < 0.05). Moreover, *Synergistetes* and *Phascolarctobacterium* were negatively correlated to TRAb, which suggests that *Synergistetes* and *Phascolarctobacterium* play a protective role in GD by protecting the thyroid gland (Chen et al., 2021). Changes in gut microbiota composition have also been reported in other autoimmune diseases and generally involve Proteobacteria, Actinobacteria, and *Lactobacillus* (Xu et al., 2019).

Some specific intestinal bacteria also play a crucial role in the development of GD. One study showed that the abundance of the genus *Prevotella* was significantly higher in GD patients (Ishaq et al., 2018). The incidence of AITD was significantly greater in patients with *Helicobacter pylori* infection (Arslan et al., 2015). Similarly *Yersinia enterocolitica* infection may play a role in the etiology of GD in Turkey (Corapcioglu et al., 2002).

The gut microbiota have functions to regulate Th17 cells (Duan and Kasper, 2011; Kim et al., 2016). Similarly, the presence of gut microbiota can increase the induction of Tregs, and the butyrate produced by gut microbiota enhances Tregs differentiation and maturation (Furusawa et al., 2013). Propionic acid is an important metabolite of *Bacteroides fragilis* (*B. fragilis*). Su et al. found that *B. fragilis* regulates Th17/Treg balance via the propionic acid-mediated pathway (Su et al., 2020). We can infer that a disruption of the gut microbiota result in abnormal secretion of Th17 and Tregs, which leads to the development of GD.

Gut microbiota disorders may induce the abnormal expression of Th17 and Treg cells, which in turn influence the occurrence and development of GD. Therefore, in-depth studies on gut microbiota disorders may provide new approaches for the treatment and prevention of this disease (Supplementary Table S3).

## Future Perspectives and Treatment of GD

Recently, Chen et al. observed that Tregs dysregulation and the expansion of pathogenic T cell clones might be involved in the long-lasting phase of GD via upregulating chemotaxis or inflammation response, and proposed a treatment with ATDs combined therapies, especially those aimed at improving Tregs frequencies or targeting specific expanded pathogenic TCR clones (Chen et al., 2021).

ATDs combination therapy provides new ideas and methods for the treatment of GD. In particular, targeting the Th17/Tregs balance may be a new approach for treating GD in the future.

## Conclusion

**FIGURE 1:**
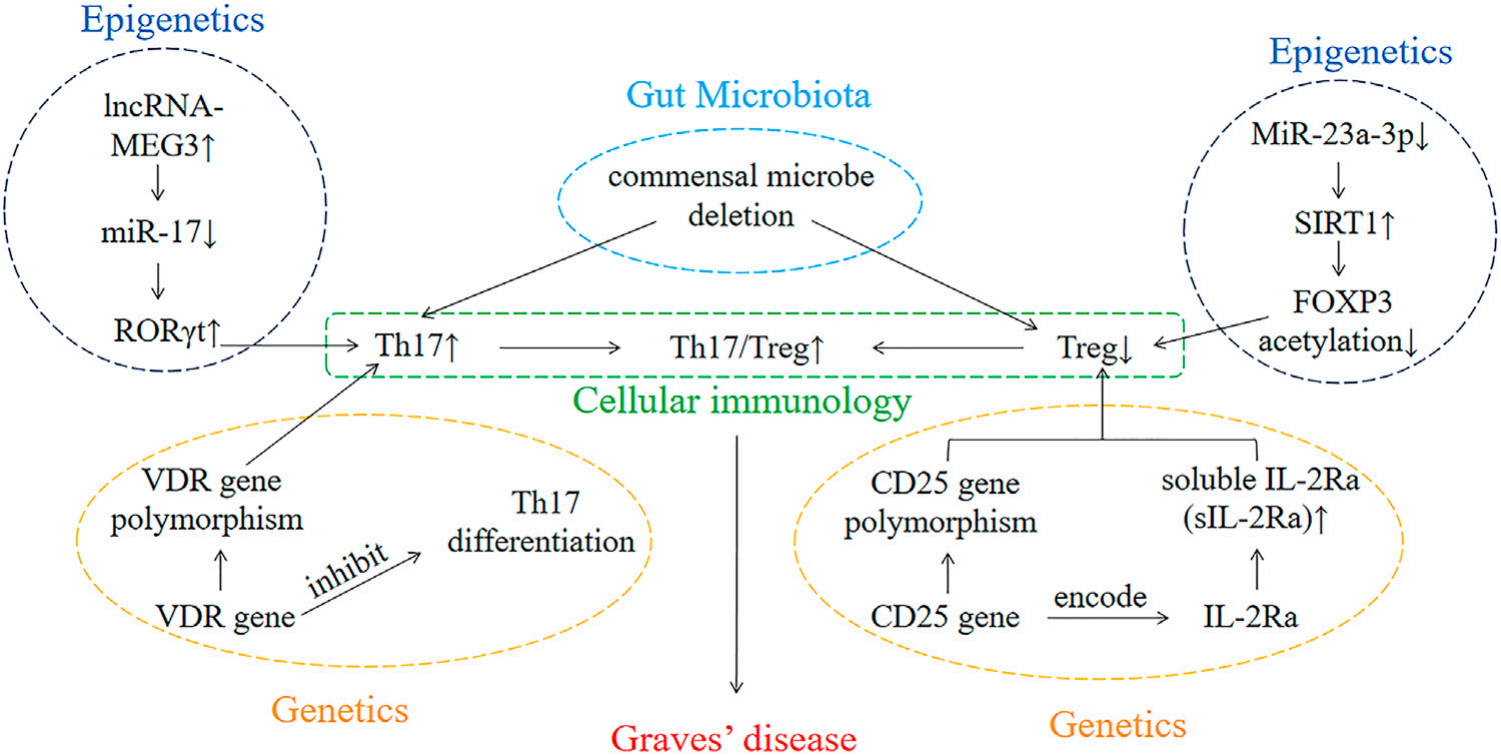
The interaction of genetics, epigenetics, cellular immunology, and gut microbiota involved in the development of GD.

Overall, genetics, epigenetics, cellular immunology, and gut microbiota play crucial roles in the pathogenesis of GD (Figure 1). In parallel, these factors also influence the occurrence and development of other autoimmune diseases. Consequently, if we elucidated the pathogenesis of GD, then it will also provide new ideas in the study of the pathogenesis of other autoimmune diseases and facilitate in the development of methods for the treatment of other autoimmune diseases.

GD is an organ-specific autoimmune disease, in which the T cell immune imbalance of patients not only participates in the occurrence and development of the disease, but also affects the function of the thyroid gland. However, no studies have explained why the changes of PBMC only affect the thyroid gland, and further studies should focus on this issue.

In this Review, we found that the similarities between GD patients and mouse models (Supplementary Figure S1). This discovery helps us to have a clearer comprehension of GD, thereby providing a direction for future research.

Although we have some understanding of the pathogenesis of GD through the Review, our comprehension of the etiology of GD is still limited. In the future, we need to further investigate the factors mentioned above in order to find potential targets for GD therapy.
