# Supplementary material for: Genetics, Epigenetics, Cellular Immunology, and Gut Microbiota: Emerging Links With Graves’ Disease
Source: Front Cell Dev Biol. 2022 Jan 4;9:794912. doi: 10.3389/fcell.2021.794912 (PMC8765724; doi:10.3389/fcell.2021.794912)
Supplement: Supplementary file 1 [file DataSheet1.doc]

**
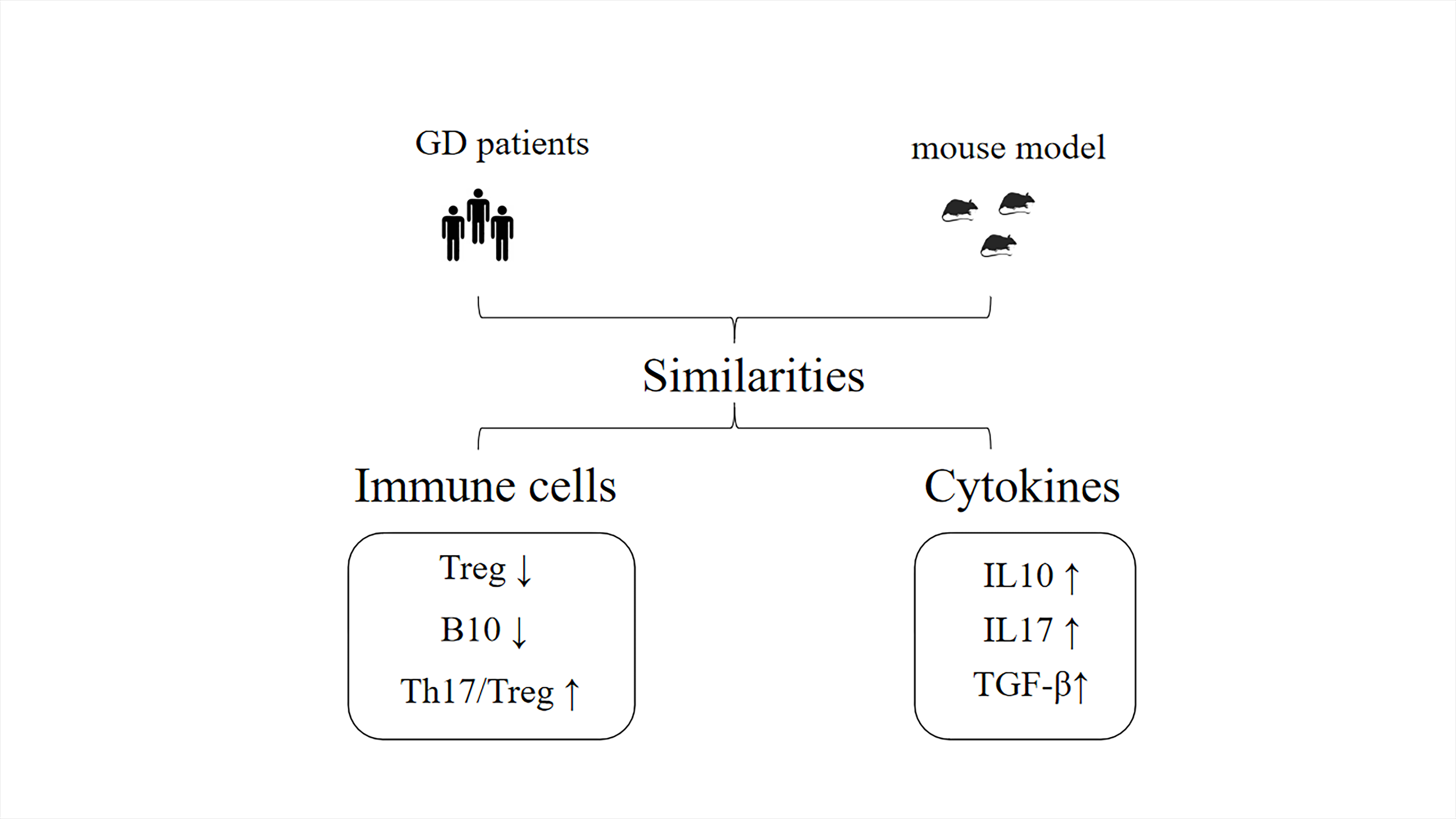
**

**Fig. S1. The similarities between GD patients and mouse mode**

**Table S1 Genetic alterations described in GD patients.**

| **Gene/Variant** | **Population** | **Cases/Controls** | **Frequency,% (cases vs. controls)** | **OR (95% CI)** | **P value** | **Reference** |
| --- | --- | --- | --- | --- | --- | --- |
| *HLA*-A*68 | Iranian | 80/180 | 15.62 vs. 4.18 | 4.23 (2.17-8.28) | 0.004 | Mehraji et al., 2017 |
| *HLA*-A*33 | Iranian | 80/180 | 0 vs. 6.98 | - | 0.011 | Mehraji et al., 2017 |
| *HLA*-B*08 | Iranian | 80/180 | 8.81 vs. 2.51 | 3.72(1.57-8.78) | 0.030 | Mehraji et al., 2017 |
| *HLA*-B*46 | Koreans | 73/159 | 24.7 vs. 5.7 | 5.455(2.974-10.004) | < 2.4×10^-8^ | Cho et al., 2011 |
| *HLA*-A*02 | Koreans | 41/159 | 48.8 vs. 33.3 | 1.905(1.165-3.115) | < 0.014 | Cho et al., 2011 |
| *HLA*-Cw*01 | Koreans | 73/159 | 37.7vs. 20.8 | 2.208(1.500-3.550) | < 0.00016 | Cho et al., 2011 |
| *HLA*-Cw*07 | Koreans | 41/159 | 2.4 vs. 14.8 | 0.144(0.034-0.607) | < 0.001 | Cho et al., 2011 |
| *HLA*-DQB1*0201 | Iranian | 80/180 | 15.62 vs. 26.81 | 0.50 (0.31-0.82) | 0.04 | Mehraji et al., 2017 |
| *HLA*-DQA1*0201 | Iranian | 80/180 | 6.25 vs.15.8 | 0.37 (0.19-0.76) | 0.045 | Mehraji et al., 2017 |
| *HLA*-DRB1*07 | Koreans | 41/159 | 1.2 vs. 1.7 | 0.128 (0.017-0.954) | < 0.015 | Cho et al., 2011 |
| *HLA*-DRB1*08 | Koreans | 41/159 | 30.5 vs. 11.3 | 3.436 (1.915-6.163) | < 5.6×10^-5^ | Cho et al., 2011 |
| *HLA*-DRB1*01 | Romanian | 77/445 | 3.89 vs. 16.4 | 0.20 (0.05-0.70) | 0.007 | Martin et al., 2014 |
| *HLA*-DRB1*03 | Romanian | 77/445 | 41.55 vs. 17.75 | 3.29 (1.90-5.68) | < 0.0001 | Martin et al., 2014 |
| *HLA*-DRB1*11 | Romanian | 77/445 | 42.85 vs. 30.56 | 1.70 (1.00-2.87) | 0.045 | Martin et al., 2014 |
| *HLA*-DRB1*15 | Romanian | 77/445 | 10.38 vs. 21.34 | 0.42 (0.18-0.95) | 0.038 | Martin et al., 2014 |
| ***CTLA*4/**rs231775 | Chinese | 260/248 | 74.81 vs. 66.13 | 1.521(1.159-1.996) | 0.002 | Chen et al., 2018 |
|  | Kashmiri | 135/150 | 48.18 vs. 27.67 | 1.85(1.30-2.63) | < 0.001 | Shehjar et al., 2020 |
| ***CTLA*4/**rs3087243 | Chinese | 260/248 | 26.92 vs. 37.70 | 1.615(1.137-2.294) | 0.007 | Chen et al., 2018 |
|  | Kashmiri | 135/150 | 46.67 vs. 28.33 | 2.21(1.56-3.13) | < 0.001 | Shehjar et al., 2020 |
|  | Brazilian | 282/308 | 39.72 vs. 21.75 | 2.593(1.630-4.123) | < 0.0001 | Bufalo et al., 2021 |
| ***PTPN*22/**rs2476601 | Caucasians | 171/200 | 4 vs. 1 | 4.23(0.87-20.62) | < 0.05 | Zhebrun et al., 2011 |
|  | Poland | 142/160 | 20 vs. 10 | 2.13(1.2-4.0) | 0.009 | Rydzewska et al., 2018a |
| ***FoxP*3/**rs3761548 | Kashmiri | 135/150 | 74.81 vs. 46.00 | 3.48(2.05-5.92) | < 0.001 | Shehjar et al., 2018 |
| ***FoxP*3/**rs3761549 | Kashmiri | 135/150 | 22.23 vs. 5.33 | 5.62(2.43-13.00) | < 0.001 | Shehjar et al., 2018 |
|  | Caucasian | 109/75 | 15 vs. 7 | 2.174(1.059-4.464) | 0.03 | Bossowski et al., 2014 |
| ***CD*25/**rs2104286 | Chinese | 650/1300 | 72.261 vs. 62.615 | 1.636(1.330-2.012) | 8.772×10^-6^ | Du et al., 2021 |
| ***CD*25/**rs41295061 | Russian | 1474/1609 | 9.7 vs. 7.0 | 1.43(1.19-1-72) | 0.00017 | Chistiakov et al., 2011 |
| ***CD*25/**rs11594656 | Russian | 1474/1609 | 7.9 vs. 5.4 | 1.54(1.14-2.07) | 0.0053 | Chistiakov et al., 2011 |
| ***IKZF*3/**rs2941522 | Chinese | 604/814 | 33.53 vs. 29.42 | 1.21(1.03-1.42) | 0.02 | Li et al., 2018 |
| ***IKZF*3/**rs907091 | Chinese | 604/814 | 33.94 vs. 29.12 | 1.25(1.07-1.47) | 0.006 | Li et al., 2018 |
| ***IKZF*3/**rs1453559 | Chinese | 604/814 | 30.55 vs. 25.98 | 1.25(1.06-1.48) | 0.007 | Li et al., 2018 |
| ***IKZF*3/**rs12150079 | Chinese | 604/814 | 23.43 vs. 19.17 | 1.29(1.08-1.55) | 0.006 | Li et al., 2018 |
| ***IKZF*3/**rs2872507 | Chinese | 604/814 | 30.46 vs. 26.61 | 1.27(1.08-1.50) | 0.004 | Li et al., 2018 |
| ***BAFF*/**rs2893321 | Chinese | 223/243 | 35.40 vs. 43.90 | 0.70(0.54-0.92) | 0.009 | Lin et al., 2016b |
| ***BAFF*/**rs4000607 | Chinese | 444/447 | 10.36 vs. 6,04 | 1.80(1.10-2.95) | 0.019 | Lane et al., 2019 |
| ***CD*40/**rs1883832 | Chinese | 196/122 | 64.5 vs. 53.7 | 1.57(1.13-2.17) | 0.008 | Wang et al., 2017b |
|  | Japanese | 61/42 | 44.5 vs. 28.9 | 1.972(1.058-3.673) | 0.031 | Inoue et al., 2012 |
| ***BACH*2/**rs2474619 | Chinese | 8882/9431 | 67.0 vs. 64.0 | 1.13(1.08-1.18) | 3.28×10^-8^ | Liu et al., 2014 |
| ***FAM*167*A-BLK*/**rs2618431 | Chinese | 624/797 | 51.282 vs. 45.797 | 1.246(1.010-1.537) | 0.04 | Song et al., 2018 |
| ***TSHR*/**rs4411444 | Japanese | 180/111 | 59.4 vs. 36.0 | 2.602(1.596-4.241) | 0.000105 | Fujii et al., 2017 |
| ***TSHR*/**rs2300519 | Japanese | 180/111 | 53.3 vs. 39.6 | 1.740(1.077-2.812) | 0.0228 | Fujii et al., 2017 |
| ***TSHR*/**rs4903961 | Japanese | 62/48 | 78.2 vs. 63.5 | 2.061(1.136-3.739) | 0.0166 | Fujii et al., 2017 |
| ***TSHR*****/**rs179247 | Japanese | 180/111 | 52.8 vs. 36.0 | 1.984(1.221-3.224) | 0.0052 | Fujii et al., 2017 |
|  | Poland | 142/160 | 47.0 vs.37.0 | 1.51(1.0-2.3) | 0.016 | Rydzewska et al., 2018a |
| ***TG*/**rs2069550 | Chinese | 436/316 | 63.1 vs. 53.4 | 1.49(1.10-2.02) | 0.01 | Gu et al., 2010 |
| ***TG*/**rs2294025 | Chinese | 9757/1062 | 21.6 vs.19.2 | 1.16(1.11-1.22) | 1.52×10^-9^ | Xuan et al., 2019 |
| ***TG*/**rs7005834 | Chinese | 9757/1062 | 85.6 vs.83.7 | 1.16(1.10-1.22) | 1.62×10^-7^ | Xuan et al., 2019 |
| ***TG*/**rs2703013 | Japanese | 131/89 | 21.5 vs. 36.1 | 0.484((0.253-0.925) | 0.0283 | Mizuma et al., 2017 |
| ***T******G*/**rs2958692 | Japanese | 50/40 | 61.1 vs. 39.7 | 2.382(1.281-4.432) | 0.0055 | Mizuma et al., 2017 |
| ***VDR*/**rs7975232 | Chinese | 417/301 | 30.34 vs. 25.42 | 1.278(1.010-1.617) | 0.041 | Meng et al., 2015 |
|  | Japanese | 139/76 | 72.8 vs. 62.7 | 1.594(1.035-2.457) | 0.034 | Inoue et al., 2014 |
|  | Chinese | 650/1209 | 12.31 vs.6.97 | 1.87(1.36-2.58) | 3.45×10^−4^ | Zhou et al., 2021 |
| ***Bcl-*2/**rs1800477 | Japanese | 264/79 | 97.0 vs. 92.4 | 2.630(1.217-5.685) | 0.011 | Inoue et al., 2016 |
| ***TNFR*2/**rs1061622 | Japanese | 160/87 | 18.5 vs. 11.0 | 1.827(1.014-3.291) | 0.038 | Inoue et al., 2016 |
| ***RNASET*2/**rs9355610 | Chinese | 701/938 | 51.2 vs. 46.1 | 1.225(1.063-1.412) | 0.005 | Chen et al., 2015b |

**Table S2 Epigenetic alterations described in GD patients**

| **Classify** | **Gene/Histone/MiRNAs** | **Epigenetic alterations** | **Sample type** | **Sample size (cases/controls)** | **References** |
| --- | --- | --- | --- | --- | --- |
| **DNA methylation** | ***DNMT*1** | hypomethylation | blood cells | 51/39 | Guo et al., 2018 |
|  | ***TSHR*** | hypermethylation | CD4^+^ and CD8^+^ T cells | 38/31 | Limbach et al., 2016 |
|  | ***ICAM*1** | hypomethylation | blood sample | 40/40 | Shalaby et al., 2019 |
|  | ***IL*2*RA*** | hypomethylation | peripheral blood leukocytes | 9/55 | Kyrgios et al., 2020 |
|  | ***TNFA*** | hypermethylation | peripheral blood leukocytes | 52/29 | Morita et al., 2018 |
| **Histone modifications** | **H3K4me3, H3K27ac** | histone hypermethylation | CD4^+^ and CD8^+^ T cells | 38/31 | Limbach et al., 2016 |
|  | **H3K9** | histone hypomethylation | PBMCs | 68/32 | Yan et al., 2019 |
|  | **HDAC1, HDAC2** | deacetylases | PBMCs | 30/20 | Yan et al., 2015 |
| **MiRNAs** | **miR-346** | downregulated | CD4^+^ T cells | 23/24 | Chen et al., 2015a |
|  | **miR-181d** | upregulated | CD4^+^ T cells | 23/24 | Chen et al., 2015a |
|  | **miR-154, miR-376b, miR-431** | downregulated | PBMCs | 41/35 | Liu et al., 2012 |
|  | **miR-23b-5p, miR-92a-39** | downregulated | serum | 7/7 | Hiratsuka et al., 2016 |
|  | **miR-Let-7g-3p, miR-339-5p** | upregulated | serum | 7/7 | Hiratsuka et al., 2016 |
|  | **miR-16, miR-22,**  **miR-375, miR-451** | upregulated | serum | 17/20 | Yamada et al., 2014 |
|  | **miR-23a-3p** | downregulated | CD4^+^ T cells | 32/20 | Zhang et al., 2019 |
|  | **miR-122-5p,miR-16-1-3p, miR-221-3p, miR-762** | upregulated | plasma | 49/39 | Yao et al., 2019 |
|  | **miR-144-3p** | downregulated | plasma | 49/39 | Yao et al., 2019 |
|  | **miR-22, miR-183** | upregulated | thyroid tissue | 22/15 | Qin et al., 2015 |
|  | **miR-101, miR-197, miR-660** | downregulated | thyroid tissue | 22/15 | Qin et al., 2015 |

**Table S3** **Gut microbiota involved in the develpoment of GD**

| **Classify** | **Gut microbiota** | **Abundance** | **References** |
| --- | --- | --- | --- |
| **Phyla** | Firmicutes | Lower | Ishaq et al., 2018;Chang et al., 2021 |
|  | Bacteroidetes | Higher | Ishaq et al., 2018; Chang et al., 2021 |
|  | Proteobacteria | Higher | Ishaq et al., 2018 |
|  | Actinobacteria | Higher | Chang et al., 2021 |
| **Genus** | Lactobacillus | Higher | Yang et al., 2019; Chen et al., 2021 |
|  | Oribacterium, Aggregatibacter, Mogibacterium | Higher | Yang et al., 2019 |
|  | Veillonella, Streptococcus | Higher | Chen et al., 2021 |
| **Bacteria** | Prevotella | Higher | Ishaq et al., 2018 |
|  | Helicobacter pylori | Higher | Arslan et al., 2015 |
|  | Yersinia enterocolitica | Lower | Corapcioglu et al., 2002 |

**Table S4** **Abbreviations**

| **Abbreviations** | **Full name** |
| --- | --- |
| **AITDs** | autoimmune thyroid diseases |
| **ATDs** | antithyroid drugs |
| **B10** | IL-10-producing B |
| **B. Fragilis** | Bacteroides fragilis |
| **CTLA4** | cytotoxic T lymphocyte-associated antigen-4 |
| **flCTLA4** | full-length CTLA4 |
| **sCTLA4** | soluble CTLA4 |
| **DNMTs** | DNA methyltransferases |
| **GD** | Graves’ disease |
| **HLA** | human leukocyte antigen |
| **H3K4me3** | trimethylation of the Lys-4 residue of histone 3 |
| **H3K27ac** | histone 3 lysine 27 acetylation |
| **ICAM1** | intercellular adhesion molecule 1 |
| **IFN-γ** | interferon-γ |
| **IL-2RA** | interleukin-2RA |
| **LncRNAs** | Long non-coding RNAs |
| **LYP** | lymphoid protein tyrosine phosphatase |
| **PBMCs** | peripheral blood mononuclear cells |
| **PTPN22** | Protein Tyrosine Phosphatase Non-Receptor Type 22 |
| **RA** | rheumatoid arthritis |
| **RAI** | radioiodine |
| **RORγt** | retinoid-related orphan receptor gamma t |
| **Tfh** | follicular helper T |
| **cTfh** | circulating Tfh |
| **TgAbs** | thyroglobulin antibodies |
| **TGF-β** | transforming growth factor beta |
| **Th17** | T helper 17 |
| **TPOAbs** | thyroid peroxidase antibodies |
| **TRAbs** | thyroid-stimulating hormone receptor antibodies |
| **Tregs** | regulatory T cells |
| **TSHR** | thyroid-stimulating hormone receptor |
